# Erratum to: Excessive Parallelism in Protein Evolution of Lake Baikal Amphipod Species Flock

**DOI:** 10.1093/gbe/evaa256

**Published:** 2021-04-11

Genome Biol. Evol. 12(9):1493–1503; doi:10.1093/gbe/evaa138

In the originally published version of this manuscript, important author corrections were inadvertently omitted and listed in this erratum.

Upon the original publication, the middle initials for co-authors Mikhail I Schelkunov, Tatyana V Neretina, Alexey S Kondrashov and Lev Y Yampolsky was omitted from the author list.

Upon the original publication, the following text should be added to the Acknowledgment section: “Laboratory work was partially funded by the Russian Foundation for Basic Research (grant # 18-05-60158).”

Upon the original publication, the following link to Github should be added for reader reference: https://github.com/ValeBurskaia/P-test

These have now been corrected online. The publisher apologizes for the error.

